# Has the Art of Filling Teeth Progressed in Fifty Years?

**Published:** 1903-04

**Authors:** 


### Editorial.

### HAS THE ART OF FILLING TEETH PROGRESSED IN FIFTY YEARS?

To the majority of dental workers in this the beginning of the twentieth century this query will seem absurd, and, given an interpretation as it stands, it could be answered very decidedly in the affirmative. This answer would, however, merely show a very superficial knowledge of the history of this portion of dental work.

In order to comprehend the steps that have been taken to reach present standards of work, the student of dental history must go back to the very beginning of the nineteenth century, or even to the middle of the eighteenth. The fact that reasonably good operations were performed from the period named extending to 1840 is certain. While the personal records of the prominent men of that lengthened time demonstrate satisfactory efforts in the direction of preserving teeth, yet the years comprising the first half of the nineteenth century must be regarded, as far as this country was concerned, as the formative period, the fallow age of dentistry. This dark period was illumined here and there by brilliant intellects, but these only served to intensify the prevailing professional gloom. The renaissance occurred about 1840, and from that time to the present the work has steadily advanced, gradually moulding dentistry into a profession.

It is not surprising that the process of filling teeth, or, as our English co-workers would say, "stopping teeth," has from the first been a leading factor in the onward movement. The general supposition has been that as this advanced the entire curriculum of study moved with it. Whether this has been true must be decided by each individual mind, for facts are wanting to demonstrate its truth.

The writer entertains some views in regard to the filling of teeth that may not be in harmony with accepted opinions. While it is recognized that this is a part of dentistry of vital importance, and that much thought and practice has been given to its development, it must be remembered that in the last analysis it is simply a mechanical substitution for lost parts and at the best is very imperfect. Strictly speaking, it ranks with the manufacture of artificial limbs, and, like the latter, serves but a temporary purpose if the life of the tooth be considered, yet the value of this temporary protection can hardly be over-estimated or overstated.

The question that more nearly interests us to-day is, Has the art of filling teeth really made any great advance in fifty years? To answer this the investigation must be concentrated in the period running from 1840 to 1855. To those personally familiar with any portion of this time it will be a useless waste of words to tell them that the operator of that period did excellent work with his non-cohesive foil. The names of Townsend, Arthur, Westcott, Rich, Taft, Dwinelle, and a host besides come naturally before the mind, and when it is said, as it has been by some, that the present work is far superior, the statement will not bear examination in the light of personal experience. It is true non-cohesive gold was used, and it is equally true that contour work was not, as a rule, attempted, but that teeth were not saved by this process is not true, nor is it a fact that the operations were poorly done. The writer has never seen better or more artistic work done than that performed by Dr. Elisha Townsend before his classes, and that without any of the modern appliances. His preparation of cavities, as described with great minuteness in his lectures, was very nearly upon the same mechanical lines so strongly advocated to-day by extension for prevention advocates. He even had the "step" so much regarded as of great value and entirely new in this twentieth century. His filling of root-canals with gold was perfection itself, but is now a lost art with the majority, for the excellent reason that gold is no longer used in filling root-canals. It serves, however, to show that the skill then acquired has not been exceeded by the present school of operators.

While much has been said in favor of cohesive gold as a preserver of tooth structure, and its value, properly used, is recognized by the writer, it must be evident to those who have passed through all the stages of progress from 1840 to 1903 that non-cohesive gold did for the preservation of teeth more than cohesive gold has done with an equal number of operators. Limitation of space prevents argument in support of this, but the fact must be self-evident that close adaptation to tooth walls is a prime essential to the salvation of teeth, and with cohesive gold this is only accomplished by the greatest care in packing, while a minimum amount of skill will insure non-leakage with non-cohesive gold. Again, the manipulator of cohesive gold will begin at the cervical wall in approximal cavities with his mallet and carry his gold in the same manner against frail lateral walls. The result of this repeated jarring means cracks in the thin enamel, and through osmotic action decay is an assured finality to such an unreasonable method. The non-cohesive operator of 1840 was not troubled with caries penetrating his filling at the cervical border or other frail surfaces, for his hand-pressure never produced cracks.

Persons familiar with histological work can have no difficulty in understanding why the cervical walls decay and why lateral walls eventually leak under cohesive gold. The question, Have we progressed? comes, then, with startling force with these facts thoughtfully considered.

It is unquestionably true that when Arthur, in 1855, introduced cohesive gold, it was the beginning of more artistic excellence in operative procedures. The lack of system under non-cohesive instruction was the great difficulty in teaching the process. It can be truthfully said that it was practically impossible to reduce this to a system. Each man had his own ideas, and the undergraduate was expected to evolve from these a method best suited to his abilities. When, therefore, cohesive gold was introduced the dental mind was not prepared to properly develop the process, and it took ten years, from 1855 to 1865, to remodel instruments and perfect skill in the use of this gold in the presence of moisture, for it must be remembered that the rubber dam was not introduced by Barnum until 1864, and its use was altogether experimental for several years thereafter. It required appliances to make it effective, and these had to be invented. The dental engine was an unknown force, separators were not, and matrices were unknown; in fact, the operator was forced to contend with all the environing difficulties then present, with no assistance from any of these aids that subsequently made the filling of teeth a comparatively easy task. Yet notwithstanding this, the ten years of novitiate in the use of a new form of gold began to bear rich fruitage, and some of the best work the writer has seen was turned out in these earlier days. It must be remembered that the previous training in the working of gold and silver helped materially in perfecting this work.

It is a common thought that the fillings made prior to the advent of Webb were of an inferior quality. No greater mistake can be made than to accept this as a fact. Webb, through his mechanical ability and persistent clinical demonstrations, impressed the dental mind of his day more forcibly than others who had preceded him, but that his work was superior to the best then known cannot truthfully be maintained. He was an enthusiast, and probably never fully satisfied his own ideals. The writer has known him to spend from early morning until late in the afternoon finishing two fillings. This, theoretically, may be artistic, but it is not practical, and Dr. Webb fell a martyr to his own high conception of duty. That he succeeded in impressing many minds must remain to his high honor, and the influence thus left has been to the maintaining and upbuilding of this branch of dental mechanical art, but let it not be forgotten that he was one among many, and to no one man can the entire credit be given of perfecting the art of filling teeth in this country.

Has this art, therefore, really made any progress since 1855? The superficial observer will be surprised at the question, and yet if the writer were to answer by the assertion, it has not, the astonishment would probably be intensified. The basic methods of filling teeth, shaping of cavities upon the mechanical principles to retain the filling, have not materially changed in fifty years, notwithstanding the earnest talk of "extension for prevention." The good men of 1850 taught the same thing, but had no catching title as a name for it. The real progress made in a half-century has been in matters of detail and the invention of appliances. With the rubber dam, clamps, separators, engine, electrical and power mallets, and the use of electrical currents, there is not a tithe of the skill required to-day to insert a filling that was then demanded. Indeed, given good gold and the appliances named, he must be a blundering mechanic who cannot make a filling worthy the best efforts of the early masters. All the talk that we hear so much of to-day regarding extension for prevention is simply throwing dust in the eyes of the modern operator to prevent his seeing the records of past history and reading therefrom that practical results, whether through the use of non-cohesive or cohesive gold, are worth more than a whole decade of theorizing.

